# Shared and unique microbes between Small hive beetles (*Aethina tumida*) and their honey bee hosts

**DOI:** 10.1002/mbo3.899

**Published:** 2019-07-04

**Authors:** Qiang Huang, Dawn Lopez, Jay D. Evans

**Affiliations:** ^1^ Honey Bee Research Institute Jiangxi Agricultural University Nanchang China; ^2^ USDA‐ARS Bee Research Laboratory Beltsville Maryland USA

**Keywords:** honey bee, metagenome, microbe, small hive beetle, virus

## Abstract

The small hive beetle (SHB) is an opportunistic parasite that feeds on bee larvae, honey, and pollen. While SHBs can also feed on fruit and other plant products, like its plant‐feeding relatives, SHBs prefer to feed on hive resources and only reproduce inside bee colonies. As parasites, SHBs are inevitably exposed to bee‐associated microbes, either directly from the bees or from the hive environment. These microbes have unknown impacts on beetles, nor is it known how extensively beetles transfer microbes among their bee hosts. To identify sets of beetle microbes and the transmission of microbes from bees to beetles, a metagenomic analysis was performed. We identified sets of herbivore‐associated bacteria, as well as typical bee symbiotic bacteria for pollen digestion, in SHB larvae and adults. Deformed wing virus was highly abundant in beetles, which colonize SHBs as suggested by a controlled feeding trial. Our data suggest SHBs are vectors for pathogen transmission among bees and between colonies. The dispersal of host pathogens by social parasites via floral resources and the hive environment increases the threats of these parasites to honey bees.

## Graphical abstract

Honey bee‐associated bacteria were identified from the small hive beetles, which may facilitate the beetle thriving in the bee hive. At the mean time, the honey bee virus colonize and replicate in SHBs, dispersion of host virus by social parasites to floral resources and hives, providing additional threats to honey bees and other insects.

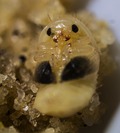

## 1 INTRODUCTION

The small hive beetle (*Aethina tumida* Murray, 1867, hereafter SHB) is a honey bee nest parasite belonging to the family Nitidulidae (sap beetles; c. 4,500 species), whose members feed mainly on decaying vegetable matter, over‐ripe fruit, or sap (Mckenna et al., 2015). Unlike other plant‐feeding beetles, SHBs can survive on fruit but thrive on resources found in honey bee colonies (Cuthbertson et al., 2013; Neumann & Elzen, 2004). SHB larvae are the most damaging stage for bee hives, by tunneling through combs and causing honey to ferment (Hood, 2004). These infestations can be destructive to wax combs, stored honey, and pollen. So far, the yeast *Kodamaea ohmeri* is known to be associated with SHBs, causing damage to the colony by fermenting stored nectar and serving as a biomarker to attract other SHBs (Benda, Boucias, Torto, & Teal, 2008). Additional symbiotic microbes associated with SHBs have not yet been described. In contrast, several symbiotic bacteria have been reported from the Asian longhorned beetle, including those that facilitate plant cell wall digestion (Scully et al., 2013), leading to insights into how these microbes impact digestion and beetle health.

Honey bee gut bacteria are dominated by nine species/clusters, some of which are likely to be involved in honey and pollen digestion, along with many low‐frequency opportunistic microbes (Kwong & Moran, 2016; Powell, Martinson, Urban‐Mead, & Moran, 2014; Raymann & Moran, 2018). As SHBs rely on food sources stored by their honey bee hosts, we predicted that SHBs might acquire honey bee‐associated microbes, which could aid in food digestion. In addition, SHBs maintain their own sets of bacteria that could aid in digestion, improving development inside the colony and when they exit as late‐stage larvae to finish development. In this study, we conducted metagenomic sequence de novo assembly to identify microbes found in larval and adult life stages of SHBs. We then confirmed those microbes using a deep RNA‐seq data set. We further conducted controlled feeding trials to determine whether candidate microbes can colonize SHBs. We have identified microbes that might facilitate the defense and development of the SHBs. We also found bee‐associated bacteria and viruses residing in SHBs. These results shed light on beetle microbe communities and help identify risks to both bees and beetles from a communal existence, as well as complex pathogen transmission routes in this ecosystem.

## 2 EXPERIMENTAL PROCEDURE

### 2.1 Beetle collection and DNA extraction

SHBs were collected from the states of Louisiana and Maryland, USA. DNA was extracted from three adult beetles for Illumina HiSeq paired‐end sequencing in 2011. Additionally, DNA was extracted from 150 SHB larvae for PacBio sequencing in 2014. These two data sets are not related, and the sequencing was conducted at the University of Maryland. These non‐sterile adult and larval small hive beetles were scrutinized to identify microbes shared with bees, microbes unique to SHB, and microbes picked up from the hive or external (soil) environment. These two sets of DNA sequencing reads were previously used to assemble the SHB genome (https://www.ncbi.nlm.nih.gov/assembly/GCF_001937115.1/). For detailed DNA extraction and sequencing protocol, see Evans et al. (2018. Due to extremely deep sequence coverage (over 500X SHB genome coverage), we were able to accurately explore the microbial community associated with SHBs. Pooled, equimolar RNA sequencing reads of eggs, larvae, and adult beetles were previously used to construct the SHB transcriptome (over 500x SHB transcriptome coverage, as described in Tarver et al., 2016). This RNA resource was used to assess the transcriptional activity of these microbes in SHB. Both DNA and RNA sequences were previously deposited at NCBI‐Bioproject PRJNA256171.

### 2.2 Metagenomic analysis of beetle‐associated microbes

**Figure 1:**
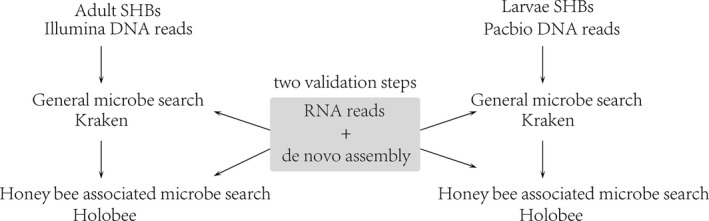
Using three independently sequenced SHB samples to identify the associated microbes, adult and larval beetle DNA reads were first aligned to all sequenced microbes genomes using Kraken and validated with RNA sequencing reads. Then, the adult and larvae DNA reads were aligned to HoloBee database and again validated with RNA sequencing reads. The adult DNA reads were de novo assembled, and the contigs were aligned to Embl, Unigene, Est, Gss, Htc, Pat, Refseq, Htg, and Tst databases to further validate the species/gene origin of the contigs

**Figure A1:**
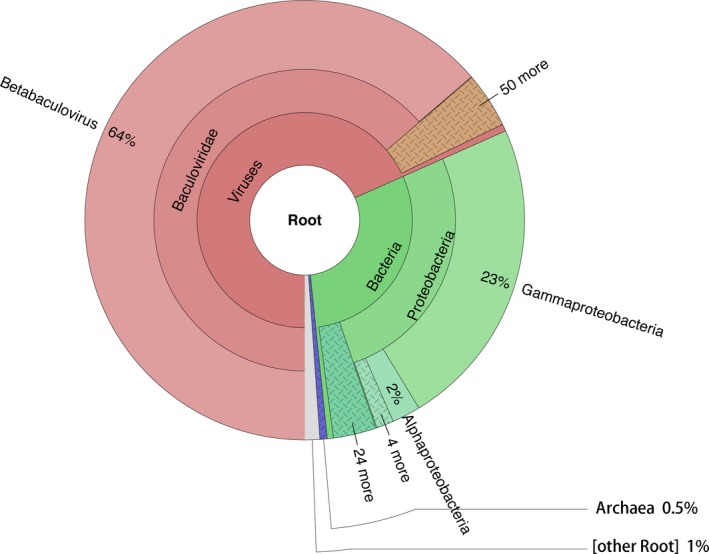
Taxonomic distributions of classified microbes associated with small hive beetle eggs, larvae, and adults. The total RNA was extracted from eggs, larvae, and adults, respectively, and then pooled for Illumina paired‐end RNA sequencing. Numbers refer to the proportion of classified sequencing reads

**Figure A2:**
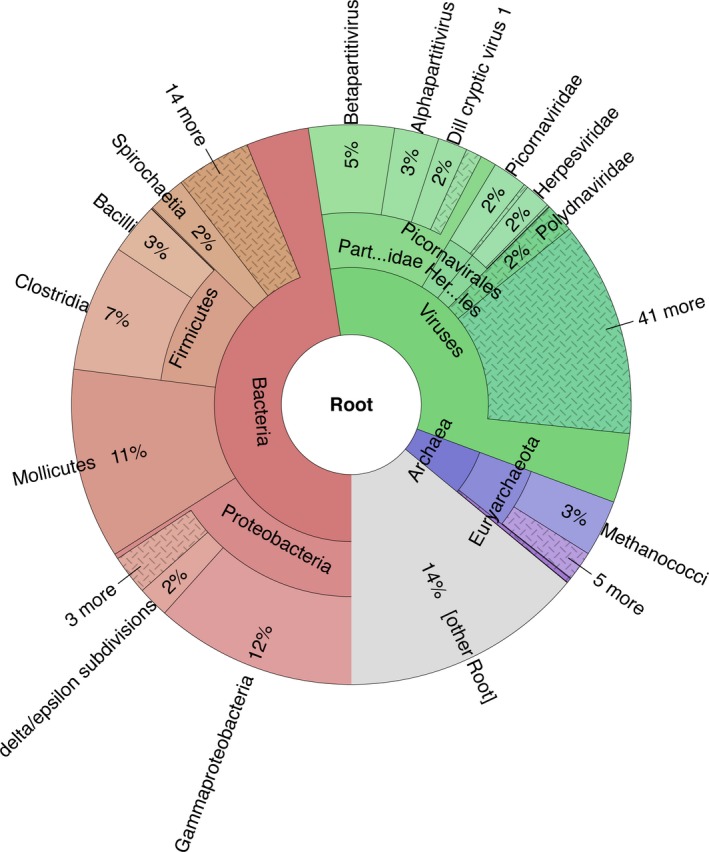
Taxonomic distributions of classified microbes associated with small hive beetle adults. Numbers refer to the proportion of classified sequencing reads

**Figure A3:**
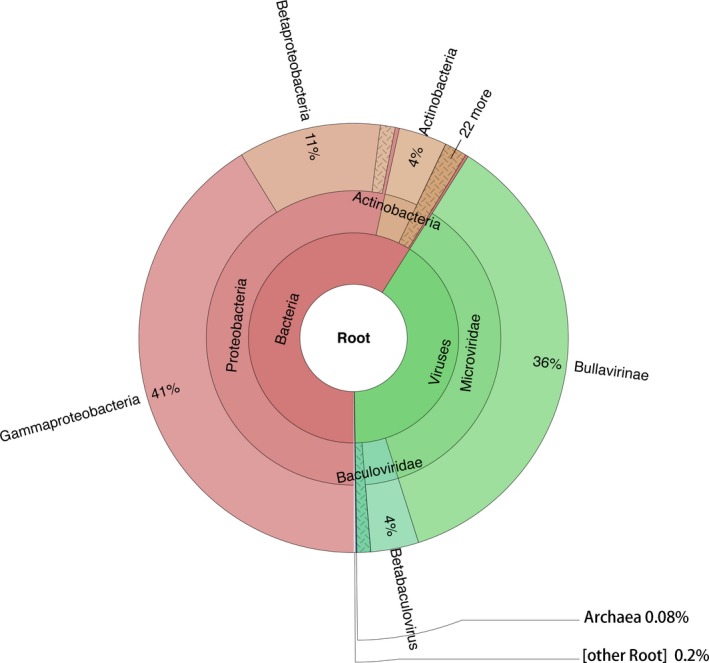
Taxonomic distributions of classified microbes associated with small hive beetle larvae. Numbers refer to the proportion of classified sequencing reads

Ilumina reads were quality checked with Fastqc (http://www.bioinformatics.babraham.ac.uk/projects/fastqc/), and PacBio reads were error corrected with Illumina reads using proovread (Hackl, Hedrich, Schultz, & Forster, 2014). DNA and RNA reads were first aligned to the SHB genome using BWA (version 0.7.13) and Tophat2 (version 2.0.13), respectively (Kim et al., 2013; Li & Durbin, 2009). Reads aligned to the SHB genome were removed. After this filtering, 96 million Illumina DNA reads, 137 million Illumina RNA reads, and 247,186 PacBio reads (~870 million nucleotides) were maintained for microbial identification. Initially, the unmapped reads were used to screen microbial species with fully sequenced genome assemblies using Kraken with standard databases, which is designed to align short sequencing reads to sequenced microbe genomes (Wood & Salzberg, 2014) (Supporting Information S1). Kraken output files were viewed using Krona (Wood & Salzberg, 2014) (Appendix Figure A1, Figure A2 and Figure A3). In order to reduce numerous false‐positive assignments of K‐mers (subset of a read) from Kraken, a de novo metagenomic assembly was produced using unmapped Illumina DNA reads by metaSPAdes assembler (version 3.10.1) with default setting (Nurk, Meleshko, Korobeynikov, & Pevzner, 2017). The assembled contigs and unmapped PacBio long reads were used to query the Embl, Unigene, Est, Gss, Htc, Pat, RefSeq, Htg, and Tst databases using BLASTN. Best hits were tallied for searches with alignment significance of *p* < 0.001. Only microbes confirmed by both Kraken and the assembled contigs were kept. In order to identify bee‐associated microbes found in SHBs, the unmapped DNA and RNA reads were aligned to the HoloBee database, a curated resource for microbes associated with honey bees (https://data.nal.usda.gov/dataset/holobee-database-v20161), using BWA (version 0.7.13) and Tophat2 (version 2.0.13), respectively. Again, candidate matches were aligned against both assembled contigs and unmapped PacBio reads to reduce false‐positive assignments (Figure 1). HoloBee‐Barcode uses a variety of markers as appropriate for each taxonomic group (Supporting Information S2). Complete 16S ribosomal RNA was used for bacteria. Barcode markers for fungi are less definitive, and ribosomal RNA internal transcribed spacer region (ITS), including ITS‐1, 5.8S, and ITS‐2, was used via Holobee database. The majority of barcodes for metazoan taxa are based on the mitochondrial locus Cytochrome C oxidase subunit I. Read counts were normalized with trimmed means of *M*‐values (TMM) using edgeR (Robinson, McCarthy, & Smyth, 2010). Over all, there are two steps to reduce false‐positive assignment of the identified microbes. First, the microbes identified from the Kraken database and Holobee database must be supported by both DNA and RNA reads. Second, the identified microbes must show significant hit when blasting the assembled de novo contigs to Embl, Unigene, Est, Gss, Htc, Pat, Refseq, Htg, and Tst databases (*p < *0.001; Supporting Information S4).

### 2.3 Verification of the identified microbes with qPCR

To further validate the accurate assignment of microbes from sequencing, a set of microbes (*Choristoneura occidentalis granulovirus, Kodamaea ohmeri*, *Deformed wing virus*, *Gilliamella apicola,* and *Snodgrassella alvi*) was selected for qPCR validation. To accomplish this, 12 adult beetles were freshly collected from apiaries near Baltimore, Maryland, in June 2018. DNA was extracted from individual beetles, and each of 3 beetles from an apiary was pooled for qPCR analysis. For detailed protocol and results, see Appendix and Supporting Information S4.

### 2.4 Colonization of honey bee‐associated microbes in SHBs

We further studied whether the selected set of microbes (*Choristoneura occidentalis granulovirus, Kodamaea ohmeri*, *Deformed wing virus*, *Gilliamella apicola,* and *Snodgrassella alvi*) can colonize small hive beetles. Accordingly, an additional 10 adult beetles were collected from the honey bee hives in Beltsville, Maryland, in September 2018. Those 10 beetles were feed with sugar water for 7 days, without introduction of any bee hive products. We hypothesize that if the microbes remained in place under this controlled diet, they can could truly colonize SHBs, instead of being merely transients collected from bee hive products. After 7 days feeding, each SHB was dissected into head thorax and abdomen sections. Then, the same body sections from five SHBs were pooled for RNA/DNA extraction, to determine specific tissue colonization of microbes. Detailed DNA extraction, RNA extraction, and qPCR protocols, along with the primers and results, are described in Supporting Information S3 and S4.

## 3 RESULTS

### 3.1 Identification of microbes from the small hive beetle

In total, 66 and 23 different microbe species were found from SHB larvae (2 archaea, 55 bacteria, and 9 viruses) and adults (22 bacteria and 1 viruses), respectively (Appendix Table A1). Of those, 14 bacteria were shared between SHB larvae and adults, including 9 putatively beneficial bacteria (Table 1). The bacteria *Gluconobacter oxydans*, *Candidatus Pantoea carbekii*, secondary endosymbiont of *Heteropsylla cubana,* and *Lactococcus lactis* were found in SHB larvae, as well as a toxin‐secreting bacterium “*Candidatus Profftella armatura*”.

**Table 1:** Symbiotic bacteria found in SHB larvae and adults and their putative functions

Microbes	Larvae	Adults	Putative functions
*Methanobrevibacter* sp.* AbM4*	D	ND	Digestion
*Candidatus Profftella armatura*	D	ND	Defensive toxin
*Candidatus Pantoea carbekii*	D	ND	Mutualists of plant‐feeding insects
*Gluconobacter oxydans*	D	ND	Synthesis of Vitamin C, D‐gluconic acid and ketogluconic acids
secondary endosymbiont of *Heteropsylla cubana*	D	ND	Insect symbiont
*Lactococcus lactis*	D	ND	Lactose digestion
*Candidatus Portiera aleyrodidarum*	D	D	Primary endosymbiont of whiteflies
*Paenibacillus mucilaginosus*	D	ND	Degrading insoluble soil minerals with the release of nutritional ions and nitrogen fixation
*Pseudomonas putida*	D	D	Breaking down aromatic or aliphatic hydrocarbons

Note

ND indicates the microbe was not found and D indicates the microbe was found.

### 3.2 Bee‐associated microbes found in the small hive beetle

As the SHB feeds on honey and pollen in honey bee colonies, these beetles are expected to receive microbes (pathogenic and symbiotic) from resident honey bees and hives. We used the Holobee database, a non‐redundant database of taxonomically informative barcoding loci for viruses, bacteria, fungi, protozoans, and metazoans associated with honey bees (https://data.nal.usda.gov/dataset/holobee-database-v20161) as a reference to identify microbial overlap between SHB and their honey bee hosts. Overall, 14 and 13 bee‐associated microbes were found in SHB larvae and adults, respectively (Table 2). Of those, seven bacteria were shared between SHB larvae and adults. We identified two additional honey bee RNA viruses in sequences derived from pooled RNA samples of all life stages.

**Table 2:** Honey bee‐associated microbes found in beetle larvae and adults, and their putative functions

Microbes	Larvae	Adult	Putative function
*Bacillus licheniformis*	D	ND	Environmental opportunist
*Citrobacter freundii*	D	D	Environmental opportunist
*Enterobacter cloacae*	D	D	Pathogenic
*Enterobacter hormaechei*	ND	D	Pathogenic
*Enterococcus faecalis*	D	ND	Pathogenic
*Escherichia coli*	D	D	Environmental opportunist
*Frischella perrara*	D	ND	Stimulating immunity
*Gilliamella apicola*	D	D	Digestion
*Klebsiella pneumoniae*	D	D	Pathogenic
*Kodamaea ohmeri*	D	ND	Honey fermentation
*Lactobacillus johnsonii*	D	ND	Digestion
*Lactobacillus kunkeei*	ND	D	Digestion
*Lactococcus garvieae*	ND	D	Pathogenic
*Lactococcus lactis*	ND	D	Environmental opportunist
*Moraxella osloensis*	ND	D	Pathogenic
*Myroides odoratimimus*	ND	D	Pathogenic
*Pseudomonas aeruginosa*	D	D	Pathogenic
*Serratia marcescens*	D	ND	Pathogenic
*Snodgrassella alvi*	ND	D	Digestion
*Staphylococcus epidermidis*	D	D	Pathogenic
*Stenotrophomonas maltophilia*	D	ND	Pathogenic
Deformed wing virus	D (RNA)		Pathogenic
Kakugo virus	D (RNA)		Pathogenic

Note

ND indicates the microbe was not found and D indicates found.

### 3.3 Verification of the microbes with qPCR

Out of the five selected microbes, only *Choristoneura occidentalis granulovirus* was not confirmed, neither from adult nor larval SHBs (Table 3, Appendix Table A2). *Kodamaea ohmeri* was consistently found in all collected SHBs, as well as a bee‐associated symbiotic bacterium *Snodgrassella alvi*. A second widespread bee symbiotic bacterium *Gilliamella apicola* was confirmed in 3 out of 4 DNA pools. The honey bee‐associated *Deformed wing virus* was confirmed in pooled RNA samples of all life stages.

**Table 3:** Verification of the microbes with qPCR. Deformed wing virus (*DWV*), *Snodgrassella alvi* (*S. alvi*), *Gilliamella apicola* (*G. apicola*), *Kodamaea ohmeri* (*K. ohmeri*), *Choristoneura occidentalis granuloviru* (*ChocGV*) were used for the assay

Collection conditions	Samples	*DWV*	*S. alvi*	*G. apicola*	*K. ohmeri*	*ChocGV*
SHB adults collected from the bee hives and directly used for DNA extraction (three samples per pool)	Adult pool #1	NA	D	ND	D	ND
	Adult pool #2	NA	D	D	D	ND
	Adult pool #3	NA	D	D	D	ND
	Adult pool #4	NA	D	D	D	ND
SHB adults collected from the bee hives and used for diet control assay and followed by RNA extraction (five samples per pool)	Abdomen pool #1	D	ND	ND	D	ND
	Abdomen pool #2	D	ND	ND	D	ND
	Thorax and head pool #1	D	ND	ND	D	ND
	Thorax and head pool #2	D	ND	ND	D	ND

Note

ND indicates the microbe was not found; D indicates found and NA represents not applicable.

### 3.4 Controlled diet analysis of SHB microbes

*Deformed wing virus* persisted in beetles fed under a controlled diet. *Gilliamella apicola* and *Snodgrassella alvi* were found in beetles collected from colonies but were absent after the controlled diet trials. The yeast *K. ohmeri* was highly abundant and constantly identified both before and after the controlled diet trials. *Choristoneura occidentalis granulovirus* was not found in beetles either before or after diet trials*.*

## 4 DISCUSSION

### 4.1 SHB unique microbes

*Candidatus Pantoea carbekii* is a known mutualism of plant‐feeding insects, which may facilitate survival and development by providing essential nutrients (Kenyon, Meulia, & Sabree, 2015). In our data, this bacterium was found in larval SHB samples, perhaps supporting the development of SHB by supplying nutrition. Protective bacteria were also found associated with SHBs. *Candidatus Profftella armatura* secretes polyketide toxins to protect plant‐feeding insect hosts from predators (Nakabachi et al., 2013), and it is conceivable that SHBs benefit from this bacterium when facing predators inside and outside the nest. For the Asian longhorned beetle, ten genera of bacteria were linked with lignocellulose and hemicellulose degradation (Geib, Jimenez‐Gasco, Carlson, Tien, & Hoover, 2009; Geib, Jimenez‐Gasco, Carlson, Tien, Jabbour, et al., 2009; Scully et al., 2013). Specific bacteria from the Asian longhorned beetle linked with plant digestion were not found in SHBs. However, SHBs might acquire additional bacteria from bee hives that play a similar role in plant cell wall digestion. In our data, colonization by the fungus *K. ohmeri* on SHB adults was verified (Table 3, Supporting Information S3). *K. ohmeri* causes honey fermentation and resulting volatiles act as a kairomone to mark the colony, attracting additional beetles (Hayes, Rice, Amos, & Leemon, 2015; Torto, Suazo, Alborn, Tumlinson, & Teal, 2005). Based on Kraken analysis, high numbers of Illumina reads were assigned to *Choristoneura occidentalis granulovirus*. However, this virus has not been found in neither de novo assembled contigs nor diet‐controlled analysis. We conclude that the k‐mer‐based assignment of Illumina reads to *Choristoneura occidentalis granulovirus* was a false positive caused by a long repetitive sequence in the assembled *Choristoneura* genome. This result demonstrates the value of following rapid heuristic searches such as Kraken with alternate forms of evidence for de novo metagenomic validation. For SHBs, the exact same microbes are not likely to be found in different life stages. Particularly, larvae must pupate in soil, quite different environmental condition compared to the bee hive. The described microbes were supported by independent data sets, reducing the chance that those microbes are falsely assigned.

### 4.2 Honey bee‐associated microbes found in SHBs

Out of the nine dominant bacteria species/clusters found in honey bees (Moran, 2015), four were found in SHBs, including three proteobacteria *Gilliamella apicola, Frischella perrara,* and *Snodgrassella alvi*, and one Firmicutes bacteria *Lactobacillus kunkeei*. The bacterium *G. apicola* facilitates pollen digestion and has a syntrophic effect with *S. alvi* that is very abundant in our study (Kešnerová et al., 2017). Acquiring this core set of honey bee bacteria arguably could help the beetle degrade pollen cell walls and digest sugars found in stored honey (Kwong & Moran, 2016). SHBs have multiple routes to acquire those bacteria, from feeding on pollen and honey, to exposure to honey bee larvae. Adult beetles also solicit food directly from their bee hosts, in the form of liquid regurgitates. Even though these symbiotic bacteria do not appear to colonize SHBs, we cannot exclude they are actively facilitating pollen and honey digestion in SHBs, as long as the beetles keep parasitizing the bee hive. Along with symbionts, SHBs host Deformed wing virus and Kakugo virus, known pathogens in honey bees. Deformed wing virus has been previously found with SHBs (Eyer, Chen, Schäfer, Pettis, & Neumann, 2009), while the others were novel to the current study. These pathogens are likely acquired orally, or via oral‐fecal transfer, as is the case with bacterial symbionts. The diet‐controlled analysis supports that Deformed wing virus can reproduce in SHBs. Furthermore, by aligning the assembled RNA sequencing contigs to the Deformed wing virus genome, both Plus/Plus and Plus/Minus matches were found. This suggests that Deformed wing virus is replicating and actively infective in SHBs, although this result should be confirmed. For one, it is conceivable that sequenced beetles have consumed honey bee eggs or larvae that themselves were infected. Regardless, SHBs are likely to act as vectors for pathogen transmission among bees and between colonies.

## CONFLICT OF INTERESTS

The authors declare no conflict of interest.

## AUTHOR CONTRIBUTIONS

JDE and QH designed the work, performed metagenomic analysis, and wrote the manuscript. DL performed qPCR validation and analyzed the data.

## ETHICS STATEMENT

None required.

## Data Availability

DNA and RNA sequencing reads were previously deposited at NCBI‐BioProject PRJNA256171.

## APPENDIX 1

### Detailed material and methods

#### DNA extraction for Illumina Hiseq‐2000 paired‐end sequencing

Genomic DNA was collected from three individual beetles from a laboratory colony at USDA, ARS Honey Bee Breeding, Genetics and Physiology Laboratory, Baton Rouge, LA, USA, at 2011. New beetles from field collections get added into the colony approximately every other month. The beetles were reared in honey combs including honey, pollen, and brood. To collect genomic DNA, the elytra were removed from each of three individual male beetles and genomic DNA was extracted using the Maxwell 16 Tissue DNA Purification Kit (Promega, Madison WI) following the manufacturer protocol and eluted using their elution buffer. Eluted gDNA was then analyzed using a Nanodrop (Thermo Scientific, Wilmington DE). The samples were not sterilized before the extraction, and the libraries were prepared without PCR amplification or polyA purification. In total, 12 libraries were prepared from the same pooled DNA following manufacturer protocol.

#### DNA extraction for Pacbio sequencing and qPCR

SHB larvae were collected from a continuous culture of small hive beetles maintained at the USDA‐ARS Bee Research Laboratory, Beltsville, Maryland, USA, at 2014. The beetles were reared in honey combs, including honey, pollen, and brood. DNA was extracted from a total of 150 s‐instar larvae in 30 groups of five larvae each. Larvae were crushed using a plastic pestle in 1 ml of freshly prepared CTAB buffer consisting of 100 mM Tris‐HCl (ph 8.0), 20 mM EDTA (ph. 8.0), 1.4 M NaCl, 2% CTAB, and 0.2% B‐mercaptoethanol. The suspension was incubated at 65°C for 60 min, with gentle mixing at 0, 20, and 40 min. Samples were centrifuged for 2 min at 14 k rpm (2081 g) in an Eppendorf microcentrifuge tube rotor. 500 μl of the supernatant was moved into a new tube containing using a wide‐bore pipette into a sterile tube containing 500 μl chloroform:isoamylalcohol (24:1). After gentle mixing by hand, tubes were centrifuged at 14 k rpm for 15 min. Approximately 400 μl of the aqueous layer was transferred into new tubes containing 250 μl cold isopropanol, followed by gentle mixing and incubation at 4°C for 30 min. Samples were centrifuged at 14 k rpm for 30 min a 4°C, and then, the supernatant was poured off. Pellets were washed with 1 ml cold 75% EtOH and centrifuged again for 2 min (14 k rpm). After the supernatant was poured off, the resulting pellets were washed in 1 ml cold 100% EtOH, centrifuged for 2 min, after which the EtOH was poured off, the pellets were spun for an additional 30 s, and the last of the wash was removed by pipette. Pellets were air‐dried for 30 min, and the resulting DNA pellet was resuspended in 50 μl ddH20. Samples were incubated for 30 min with 2.5 μl of an RNAse cocktail at 37oC, followed by gentle addition of 5 μl 7 M NaOac and 100 μl EtOH. After 30 min of incubation on wet ice, the DNA samples were spun at 12 k rpm for 30 min, washed once with 7% EtOH, dried and suspended in 20 μl ddH20. Extracts were pooled and assayed by gel electrophoresis to ensure DNA integrity and by Nanodrop (Thermo Fisher, Inc.) for quantification (180 ng/μl in 25 μl, 45 μg total DNA). The samples were not sterilized before the extraction, and the libraries were prepared without PCR amplification or polyA purification. In total, 40 SMRT cells were prepared from the same pooled DNA following manufacturer protocol.

#### Two‐step quantitative PCR for microbe validation

The below variant of qPCR is for a 96‐well plate format on the CFX96 real‐time system (Bio‐Rad) or related machines and works for both bee transcripts and pathogen targets. The primary difference over the prior protocol is that this one is initiated with cDNA generated in a non‐specific way, rather than from de novo reverse‐transcription for each viral and/or host test and control (as shown in the previous section).

1. Mix 1× SsoAdvanced™ Universal SYBR^®^ Green Supermix (Bio‐Rad) with 4 mM of each forward and reverse primer for a given target (final volume 20 μl).
2. Add 1 μl (~8 ng) of cDNA template to specific wells.
3. Use the following cycling conditions:
  - 95°C for 1 min,
  - 45 (maximum 50) cycles of:
    95°C for 5 s,
    60°C for 30 s,

Melt curve from 65–95°C at + 0.5°C/5 s increments.

**Table A1:** Identified microbes from SHB larvae and adults, normalized reads (counts per million reads) and putative function

Kingdom	Microbes	Larvae	Adults	Putative function
Archaea	*Methanobacterium lacus*	194	#N/A	
Archaea	*Methanobrevibacter* sp. AbM4	3,101	#N/A	Digestion
Bacteria	*Acinetobacter baumannii*	388	#N/A	Pathogen
Bacteria	*Arcobacter* sp. L	581	#N/A	
Bacteria	*Bacillus anthracis*	388	#N/A	Pathogen
Bacteria	*Bacillus cereus*	2,907	#N/A	Pathogen
Bacteria	*Bdellovibrio bacteriovorus*	194	#N/A	Parasite of other bacteria
Bacteria	*Blattabacterium* sp. (Blaberus giganteus)	388	#N/A	
Bacteria	*Brachyspira pilosicoli*	4,845	#N/A	Pathogen
Bacteria	*Buchnera aphidicola*	24,806	#N/A	
Bacteria	*Burkholderia pseudomallei*	#N/A	9	
Bacteria	*Burkholderia* sp. RPE64	388	#N/A	
Bacteria	*Campylobacter fetus*	775	#N/A	Pathogen
Bacteria	*Candidatus Babela massiliensis*	388	#N/A	Pathogen
Bacteria	*Candidatus Pantoea carbekii*	775	#N/A	Mutualists of plant‐feeding insects
Bacteria	*Candidatus pelagibacter* sp. IMCC9063	969	#N/A	
Bacteria	*Candidatus Phytoplasma mali*	3,295	#N/A	Pathogen, plant
Bacteria	*Candidatus Portiera aleyrodidarum*	194	9	Primary endosymbiont of whiteflies
Bacteria	*Candidatus Profftella armatura*	2,713	#N/A	Defensive toxin
Bacteria	*Clostridioides difficile*	581	#N/A	Pathogen
Bacteria	*Coxiella burnetii*	194	#N/A	Pathogen
Bacteria	*Cutibacterium acnes*	#N/A	22,901	Pathogen
Bacteria	*Cyanothece* sp. ATCC 51142	194	#N/A	
Bacteria	*Cyanothece* sp. PCC 7822	581	#N/A	
Bacteria	*Dokdonia* sp. 4H‐3‐7‐5	194	#N/A	
Bacteria	*Enterobacter cloacae*	581	14,713	Pathogen
Bacteria	*Enterococcus faecalis*	1,744	3,334	Pathogen
Bacteria	*Escherichia coli*	15,891	4,222	
Bacteria	*Flavobacteriaceae bacterium* 3519‐10	#N/A	84	Unclear
Bacteria	*Fusobacterium nucleatum*	1,550	210	Pathogen
Bacteria	*Gluconobacter oxydans*	581	#N/A	Synthesis of Vitamin C, D‐gluconic acid and ketogluconic acids
Bacteria	*Haemophilus influenzae*	7,752	157	Pathogen
Bacteria	*Helicobacter pylori*	3,682	#N/A	Pathogen
Bacteria	*Herminiimonas arsenicoxydans*	#N/A	67	Neurtal
Bacteria	*Histophilus somni*	3,101	84	Pathogen
Bacteria	*Klebsiella pneumoniae*	1,938	1,351	Pathogen
Bacteria	*Lacinutrix* sp. 5H‐3‐7‐4	388	#N/A	
Bacteria	*Lactococcus lactis*	388	#N/A	Lactose digestion, hinder pathogenic bacteria
Bacteria	*Melissococcus plutonius*	194	#N/A	European foulbrood
Bacteria	*Methylobacterium* sp. 4‐46	388	#N/A	
Bacteria	Mycoplasma conjunctivae	1,550	#N/A	Pathogen
Bacteria	Mycoplasma hyopneumoniae	101,938	#N/A	Pathogen
Bacteria	*Mycoplasma hyorhinis*	10,271	#N/A	Pathogen
Bacteria	*Mycoplasma leachii*	2,713	#N/A	Pathogen
Bacteria	*Mycoplasma mycoides*	2,132	#N/A	Pathogen
Bacteria	*Neisseria meningitidis*	969	#N/A	Pathogen
Bacteria	*Paenibacillus mucilaginosus*	2,132	#N/A	Degrade insoluble soil minerals with the release of nutritional ions and fix nitrogen
Bacteria	*Pasteurella multocida*	388	99	Pathogen
Bacteria	*Photorhabdus asymbiotica*	33,527	20	Pathogen
Bacteria	*Porphyromonas gingivalis*	#N/A	87	Pathogen
Bacteria	*Prochlorococcus marinus*	2,132	#N/A	Oxygen
Bacteria	*Proteus mirabilis*	388	#N/A	Pathogen
Bacteria	*Pseudanabaena* sp. PCC 7367	12,984	#N/A	
Bacteria	*Pseudomonas aeruginosa*	2,326	257,695	Disease
Bacteria	*Pseudomonas putida*	#N/A	673	Breaking down aromatic or aliphatic hydrocarbons
Bacteria	*Pseudomonas stutzeri*	#N/A	480	Pathogen
Bacteria	*Riemerella anatipestifer*	#N/A	233	Pathogen
Bacteria	*Rivularia* sp. PCC 7116	581	#N/A	
Bacteria	Secondary endosymbiont of Heteropsylla cubana	388	#N/A	Insect symbiont
Bacteria	*Serratia marcescens*	13,953	3	Pathogen
Bacteria	*Shigella flexneri*	2,907	594	
Bacteria	*Shigella flexneri*	2,907	594	
Bacteria	*Sorangium cellulosum*	1,163	#N/A	Soil bacteria
Bacteria	*Streptococcus agalactiae*	194	#N/A	Pathogen
Bacteria	*Streptococcus anginosus*	581	#N/A	Pathogen
Bacteria	*Streptococcus pneumoniae*	388	82	Pathogen
Virus	Anomala cuprea entomopoxvirus	581	#N/A	
Virus	Apocheima cinerarium nucleopolyhedrovirus	775	#N/A	
Virus	Gryllus bimaculatus nudivirus	581	#N/A	
Virus	Human alphaherpesvirus 3	194	#N/A	
Virus	Invertebrate iridescent virus 6	194	#N/A	
Virus	Lymphocystis disease virus—isolate China	194	#N/A	
Virus	*Megavirus chiliensis*	388	#N/A	
Virus	Orgyia leucostigma NPV	775	#N/A	
Virus	Suid alphaherpesvirus 1	194	#N/A	
Virus	Enterobacteria phage phiX174 sensu lato	#N/A	555,038	

**Table A2:** qPCR validation results for the microbes. A set of beetle (*Choristoneura occidentalis granulovirus* and *Kodamaea ohmeri*) and bee‐associated microbe (*Deformed wing virus*, *Gilliamella apicola*, *Snodgrassella alvi*, and *Melissococcus plutonius*) were further used for qPCR verification. Generally, the validation is consistent with metagenomic assembly assignment

Microbes	RNA of all life stages	DNA of larvae	DNA of adult	Primer 1	Primer 2	Reference
*Deformed wing virus*	Yes	NA	NA	GAGATTGAAGCGCATGAACA	TGAATTCAGTGTCGCCCATA	vanEngelsdorp et al. (2009)
*Gilliamella apicola*	NA	No	Yes	GTATCTAATAGGTGCATCAATT	TCCTCTACAATACTCTAGTT	Schwarz, Moran, and Evans (2016)
*Snodgrassella alvi*	NA	No	Yes	CTTAGAGATAGGAGAGTG	TAATGATGGCAACTAATGACAA	Schwarz et al. (2016)
*Choristoneura occidentalis granulovirus*	NA	No	No	TACATGGTBACNGARGA	AAYTCYTTNCCGCTCCAGTT	Krejmer‐Rabalska, Rabalski, Souza, Moore, and Szewczyk (2018)
*Kodameae ohmeri*	NA	Yes	Yes	GAGTGAAGCGGCAAAAGCTC	AACATAGACACGGTCGCCTC	Designed by J. P. Tauber (unpublished data)
*Melissococcus plutonius*	NA	Yes	No	ACGCCTTAGAGATAAGGTTTC	GCTTAGCCTCGCGGTCTTGCGTC	Evans (2006)

Note

Yes represents the primers can be amplified. No represents the primers cannot be amplified. NA represents the primers is not conducted for qPCR assay.
